# Impact of climate change on non-communicable diseases caused by altered UV radiation

**DOI:** 10.25646/11653

**Published:** 2023-09-06

**Authors:** Cornelia Baldermann, Gudrun Laschewski, Jens-Uwe Grooß

**Affiliations:** 1 Federal Office for Radiation Protection Section WR4 - Optical Radiation Neuherberg/Oberschleißheim, Germany; 2 Deutscher Wetterdienst Centre for Medical-Meteorological Research Freiburg, Germany; 3 Forschungszentrum Jülich GmbH Institute for Energy and Climate Research – Stratosphere (IEK-7) Jülich, Germany

**Keywords:** CLIMATE CHANGE, UV RADIATION, OZONE, CLOUD COVER, HEALTH, ADAPTATION

## Abstract

**Background:**

UV radiation can cause serious skin and eye diseases, especially cancers. UV-related skin cancer incidences have been increasing for decades. The determining factor for this development is the individual UV exposure. Climate change-induced changes in atmospheric factors can influence individual UV exposure.

**Methods:**

On the basis of a topic-specific literature research, a review paper was prepared and supplemented by as yet unpublished results of the authors’ own studies. The need for scientific research and development is formulated as well as primary prevention recommendations.

**Results:**

Climate change alters the factors influencing UV irradiance and annual UV dose in Germany. First evaluations of satellite data for Germany show an increase in mean peak UV irradiance and annual UV dose for the last decade compared to the last three decades.

**Conclusions:**

The climate change-related influences on individual UV exposure and the associated individual disease incidence cannot yet be reliably predicted due to considerable uncertainties. However, the current UV-related burden of disease already requires primary preventive measures to prevent UV-related diseases.

## 1. Introduction

Climate change has serious consequences for humans and the environment. Catastrophes such as floods, storms, droughts, or heatwaves clearly demonstrate this. Less obvious, but no less problematic, are the effects of climate change that can have negative consequences for humans and nature, but go unnoticed and thus insidiously worsen the situation. One of these is the climate change-induced modification in the factors influencing ultraviolet (UV) radiation exposure (in this article, UV is often used in compounds as an abbreviation for ultraviolet radiation).

UV radiation is the initiator of the body’s own vitamin D formation. At the same time, UV radiation is the main cause of skin cancer and can lead to other negative health consequences for the eyes and skin [1, 2]. UV radiation damages the genetic material and, like asbestos and ionising radiation, is classified in the highest risk group 1 as ‘carcinogenic to humans’ [3]. Especially UV-related cancers are a burden on the general welfare and cause high costs for the healthcare system [2, 4]. UV-related health damage to the eyes and skin can basically affect everyone. Children are particularly at risk [1].

According to the current state of scientific knowledge, climate change is altering the factors influencing ambient UV radiation worldwide and also in Germany and thus the UV-related human disease risk. In addition to individual UV exposure behaviour, the structures in people’s living environments that offer UV relief – such as shaded areas – play a decisive role for individual UV exposure and thus for the UV-related disease risk [2].

In this paper, the changes in ground-level UV radiation, the resulting consequences for individual UV exposure, and the associated health risk are considered and recommendations for adaptation are given. The literature search for this article was based on international reviews on the topics ‘stratospheric ozone’, ‘climate change’, ‘interactions between ozone and climate and effects on solar UV radiation’ and ‘climate change, UV exposure, human health’, among others, in the free full-text archive of biomedical and life science journal literature PubMed. In addition, reports and data services of national and international authorities and their bibliographies were used, as well as the authors’ own studies that have not yet been published. These are marked with the note ‘Author et al.; data not published’.

## 2. Development of ground-level UV radiation

### 2.1 Retrospective development of ambient UV radiation

UV radiation in the human habitat is characterised by a very strong spatial and temporal variability, which is mainly determined by the position of the sun, the thickness of the ozone layer, and cloud cover. Further influences result from the aerosol content of the air, the reflectivity of the ground, and the altitude of the location. Changes in the position of the sun are reflected in changes in UV radiation both with latitude and over the course of the day and year. The strongest absorber of UV radiation, atmospheric ozone, usually has higher concentrations at northern latitudes and in turn has an annual cycle with the highest values in March and April and the lowest values in October and November. Anthropogenic emissions of chlorofluorocarbons (CFCs) not only caused the Antarctic ozone hole, but also reduced the thickness of the ozone layer in varying degrees globally. Due to international agreements such as the Montreal Protocol in 1987 and subsequent agreements, CFC emissions have been reduced almost completely to zero compared to the 1970s and 1980s, so that chlorine pollution in the atmosphere has been declining since around the year 2000 and the thickness of the ozone layer is slowly recovering. In the case of Arctic ozone, this has not yet been verified due to the high variability [5].

#### Effects of the above-mentioned influencing factors on UV irradiance

**Figure 1:**
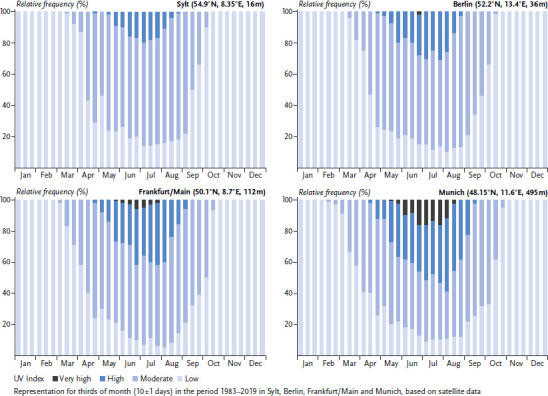
Mean relative frequency of UV Index classes at midday at four sites in Germany (indicated are latitude, longitude and altitude of the site above sea level) Source: Own representation based on Vitt et al. [7]; data basis extended to 2019 (Deutscher Wetterdienst)

How the above-mentioned influences affect ambient UV irradiance can be seen, for example, in the average frequency of occurrence of certain UV Index categories (no to low, moderate, high, very high, and extreme [6]). The UV Index (UVI) is a measure of the daily maximum of sunburn-effective (erythema-effective) UV irradiance occurring on a horizontal surface [6]. Figure 1 shows the results of the analysis of satellite data for thirds of month (periods of 10 ± 1 days) at four sites of different latitude in Germany (Sylt, Berlin, Frankfurt/Main, Munich). In the long-term average (data basis here: 1983–2019), high UV indices (UVI ≥6) occur everywhere in Germany in summer, but there are differences in how many days and which further periods are affected in spring and autumn. The affected period as well as the average proportion and the maximum number of days with high UV indices within one third of month are larger in the south than in the north of Germany. With the time division made here, high UV indices additionally occur in three thirds of month in Munich compared to Sylt. If we look at the third of month with the highest UV indices, 37% more high UV indices are recorded in Munich than in Sylt. A similar pattern can be seen with the very high UV indices (UVI ≥8), with the particularity that they typically occur extremely rarely in the very north in Sylt and therefore do not play a role in the representation of the long-term average. In Munich, ten thirds of month are affected by very high UV indices, five times as many as in Berlin, and the maximum proportion of such days within a third of month is 15% higher compared to Berlin. Low ozone events that can lead to exceptional values of the UVI are discussed later in this section.

#### Change in ambient UV radiation

**Figure 2:**
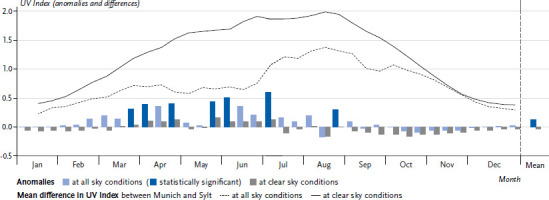
Anomalies of the UV Index (UVI) and differences in the UVI between Munich and Sylt. Columns show anomalies of the UVI in the period 2010–2019 compared to 1983–2019 per thirds of month (four-site mean). Source: Own representation based on Vitt et al. [7]; data basis extended to 2019 (Deutscher Wetterdienst)

Ambient UV radiation in Germany in the recent past differs from the long-term mean conditions. This is illustrated by a mean of the UVI anomalies of the four sites shown in Figure 1, which for each site is based on the deviations of the thirds of month during the period 2010–2019 from the respective thirds of month during the overall period 1983–2019 (Figure 2). The four-site mean in Germany shows a statistically significant increase in the values of the UVI during the period 2010–2019 compared to the long-term average 1983–2019. During the months February to July, on average, exclusively positive UVI anomalies are recorded, i.e. here the UVI had higher values on average during the last decade. The magnitude of the anomalies varies, reaching the highest values around 0.5 UVI (difference around 12.5 milliwatt per square meter (mW/m^2^) sunburn-effective UV irradiance) in early July and early June. The mean UVI anomaly in the month of June is 0.35 UVI (8.75 mW/m^2^ sunburn-effective UV irradiance). The positive UVI anomalies also occur at times of the year that are particularly significant with regard to the health consequences of UV exposure: as a rule, in spring the skin of most people in Germany is still unaccustomed to the sun and thus particularly at risk.

The period around the solar maximum in June is characterised by the highest absolute values of the UVI over the course of the year, as shown above. These results, based on satellite data, mainly reflect the effects of changes in cloud cover and ozone layer thickness. The anomalies of the UVI at clear sky conditions are, on this data basis, the result of changes in the ozone layer thickness. On average over the entire year, there is a slight (but statistically not significant) decrease in the UVI anomaly at clear sky conditions, which can be interpreted as an expression of the improving situation of the ozone layer due to the implementation of the Montreal Protocol.

Overall, the evaluations of the satellite data give first indications that ambient UV irradiance in Germany during the last decade (2010–2019) is mainly shaped by decreases in cloud cover in spring and summer. The development of the thickness of the ozone layer plays a much smaller role in comparison ([7], Laschewski et al.; data not published). The influence of longer-term aerosol changes is not represented in this data basis.

In addition to the daily maxima of the erythema-effective UV irradiance in the form of the UVI, the values summed up for all hours of the days of a year in the form of the annual UV dose are also significant, which were evaluated in analogy to the UVI for the period 1983–2019 (Laschewski et al.; data not published). The four-site mean in Germany indicates a 2% higher annual UV dose per decade when comparing the mean value of the period 2010–2019 with the long-term mean value of 1983–2019. The annual UV dose is characterised by strong variability from year to year. In 2018, a year with particularly high levels of sunshine, the mean annual UV dose of the four sites in Germany was about 13% above the long-term mean, while in 1987, a year with particularly little sunshine, it was about 16% below the long-term mean. Evaluations of the UV monitoring network data measured at ground level confirm this correlation ([8], Lorenz et al.; data not published).

The regional differences in ambient UV radiation between northern and southern Germany are considerable. The percentage changes in the UVI and the annual UV dose therefore have different regional effects. For example, based on satellite data, the annual UV dose in Munich is 29% higher than that of Sylt in the long-term average, which is primarily due to the latitude (i.e. the position of the sun) ([7], Laschewski et al.; data not published). The extent to which long-term changes in the aerosol content of the air in Germany correspond to changes in ambient UV radiation is currently unclear. For such an evaluation, quality-assured data series of ground-based measurements are necessary.

The presented findings from the analysis of satellite data for Germany ([7], Laschewski et al.; data not published), which show a 3%-per-decade higher mean value of the UVI and a 2%-per-decade higher mean annual UV dose for the period 2010–2019 compared to the overall period (1983–2019), are within the value range of the changes in UV radiation determined on the basis of ground-based measurements for several European stations [9]. In this study, measured data from stations at latitudes of Germany show changes in UV radiation ranging from -7% to +5% per decade over the last 22 years (1996–2017).

Changes in cloud cover, aerosol content of the air, and reflectivity of the ground can be identified as the main causes of long-term changes in UV radiation in most locations outside the polar regions, while changes in ozone layer thickness are less important [10]. The successful implementation of air pollution-reducing measures caused a general decrease of the aerosol content since about the mid-1980s and led to a higher radiation transmittance of the atmosphere, also in Germany [11]. As a result, an increase in ground-level UV radiation can also be assumed due to the reduction in the aerosol content of the air, which is particularly pronounced in urban areas and cannot be attributed to climate change.

#### Low ozone events

As shown above, the development of the ozone layer also plays a much smaller role in Germany in the development of ambient UV radiation than changes in cloud cover. Nevertheless, so-called low ozone events (LOEs) require special consideration, as they can lead to unexpectedly high UV exposures for a few days. There are various reasons why LOEs can occur. Firstly, weather conditions can lead to an increase in the height of the tropopause, which marks the transition from the troposphere to the stratosphere, and thus to a (reversible) displacement of part of the stratospheric ozone. This is called LOE of dynamic origin. On the other hand, it is possible that air masses from the Arctic polar vortex, in which ozone has been irreversibly depleted by chlorine chemistry, are transported to lower latitudes in spring. These LOEs of chemical origin can occur between March and mid-April.

Not all LOEs lead directly to increased ground-level UV exposure, as UV radiation can be greatly reduced by clouds or aerosols as it passes through the atmosphere. A recent study based on satellite data from 1983–2019 evaluates the occurrence of all LOEs with associated UV anomaly, i.e. on these days there is stronger ground-level UV radiation than would be recorded in cloudless conditions with normal ozone layer thickness [12]. This study shows that the annual number of LOEs of different origin is characterised by a high variability. They occur relatively rarely and not every year. LOEs of dynamic origin can occur at all months of the year. The period of greatest global ozone depletion in the 1990s is characterised by a greater frequency of LOEs. During the last two decades (1998–2019), a decreasing average number of all LOEs in a year was evident, although the annual sums of LOE-associated UV dose anomalies were unchanged on average during this period. Overall, the mean value of the annual sums of the LOE-associated anomalies of the UV dose provides a rather small contribution with less than 1/1000 of the annual UV dose. Looking at the peak loads (LOE-associated UVI anomalies), spring is the season most affected by LOEs, accounting for more than half of the cumulative peak UV loads. The skin of most people in Germany is unaccustomed to the sun at this time of year and thus particularly at risk. This results in the health relevance of LOEs occurring in spring, which do not take place every year. Within the last two decades, summer (and thus the most radiation-intensive time of the year) provides an increasing share of all LOE-associated peak loads. During the study period (1983–2019), the strongest LOE-associated increase in erythema-effective UV irradiation is achieved when LOEs occur near the annual solar peak in June or early July; with respect to the four sites in Germany considered, the maximum anomaly values for Berlin, Frankfurt, and Munich are about 45 mW/m^2^ (corresponding to 1.8 UVI). Primarily due to the different latitude (i.e. the different position of the sun), different absolute values of the UVI are associated with these anomaly maximum values in the evaluated period, ranging from UVI 8 in Berlin to UVI 9 in Munich.

### 2.2 Expected development of ambient UV radiation until the middle and end of the century

The possible future climate change developments are modelled with a number of different scenarios. An introduction to the scenarios can be found, for example, in the introductory article of this status report [13]. Assuming scenario SSP2-4.5 (radiative forcing of 4.5 W/m², the so-called middle-of-the-road scenario, in which the previous development is continued and environmental systems experience some deterioration [14]), a return to 1980 ozone column values is expected outside polar latitudes around 2035 for mid-northern latitudes (35°N–60°N) and around 2045 for the Arctic [5]. However, simulations with chemical climate models for the period 1960–2100 suggest that changes in UVB radiation at mid-latitudes in the second half of the 21^st^ century may be dominated by factors other than changes in global stratospheric ozone [15], namely a statistically significant decrease in cloud cover of 1.4% per decade. According to these calculations, UVB radiation would be projected to increase by 1.3% per decade between 2050 and 2100, although at the same time no trend in ozone layer thickness is detectable. These projections depend critically on the accurate description of clouds by the climate models, and the uncertainties in their modelling cause considerable uncertainties also for the projected changes in UV radiation.

In terms of changes in stratospheric ozone (and subsequently indirectly in terms of LOEs due to polar chemical ozone depletion), the influence of climate change leading to stratospheric cooling and an increase in stratospheric water content favours springtime ozone depletion in the Arctic polar vortex and could thus counteract recovery due to decreasing chlorine levels [5, 16]. There is a very large variability in Arctic ozone depletion from year to year. Based on modelling to date, the extent of Arctic ozone depletion is currently expected to decline in the spring until mid-century [5], with other estimates inferring a time horizon to the end of the century [16]. LOEs over Germany due to polar chemical ozone depletion, which do not occur every year, may become less frequent over the course of the century, but may continue to occur. In addition to these LOEs of chemical origin, the predominant type are LOEs of dynamic origin. It is currently unclear how the frequency of their occurrence and the associated ambient UV radiation will develop. So far, there are no known indications that would point to their fundamental absence in the coming decades. Simple extrapolations of retrospective trends would, on the one hand, suggest a decrease in ambient UV radiation due to the declining number of LOEs. On the other hand, a further decrease in cloud cover and a more frequent occurrence of LOEs in summer could make an increase in UV exposure more likely. Neither the net effect of the opposing trends nor the admissibility of the extrapolations can be reliably estimated at present. There is a need for further research.

Due to considerable uncertainties, the development of ambient UV radiation in Germany up to the middle and end of the century cannot be reliably predicted at present.

## 3. Changes in individual UV exposure and effects on UV-related diseases

The previously described climate change-related changes in the factors influencing the ground-level UV irradiance and the annual UV dose can change the individual UV exposure and thus have an effect on the risk of disease caused directly by UV radiation [1, 2, 17, 18]. Indirect health effects caused by UV radiation are, for example, the effects of increased ground-level ozone (see Breitner-Busch et al. [19] in this status report) or the negative influence of UV radiation on atopic dermatitis (see Bergmann et al. [20] in this status report), which will not be discussed in detail here.

When considering the health risks from UV radiation, both the individual UV radiation moments and the UV dose accumulated over a lifetime, the lifetime dose, must be considered due to the fact that UV radiation can immediately cause health-relevant damage and that these can accumulate over a lifetime [1, 2]. With regard to the health consequences of altered near-ground UV exposure, the increase in ground-level UV irradiance observed over the past decades [7], longer-term increased annual UV dose [8], and LOEs [21–24] must be considered individually accordingly.

UV-related skin cancer (Info box 1) is used to describe UV-related disease incidence, as there are data available that allow quantification of the disease incidence.

**Info box 1 Skin cancer**

A distinction is made between non-melanoma skin cancer and (malignant) melanoma skin cancer. Non-melanoma skin cancer includes basal cell carcinoma (basalioma) and squamous cell carcinoma (spinalioma). In the case of non-melanoma skin cancer, melanin-less skin cells (melanin = colour-giving pigment of the skin and hair) multiply uncontrollably. Non-melanoma skin cancer may be preceded by skin changes, the so-called precancerous lesions, such as actinic keratosis or Bowen’s disease. In (malignant) melanoma, melanin-containing skin cells degenerate. Both squamous cell carcinoma and melanoma can spread and metastasise.

Worldwide, an increasing incidence of skin cancer has been recorded for decades. In Germany, the incidence of non-melanoma skin cancer has quadrupled (men) to quintupled (women) in the last 30 years [2, 25]. For malignant melanoma, the incidence has approximately quadrupled since the 1970s [2, 25]. Since 2012, the incidence of malignant melanoma has declined slightly in women and remained roughly constant in men [26]. According to the extrapolations from the data of the Skin Cancer Registry Schleswig-Holstein, which in contrast to the data of the Robert Koch Institute also include in-situ melanoma and in-situ squamous cell carcinoma, about 300,000 people per year are currently newly diagnosed with skin cancer [27]. Hospital treatments of UV-related skin cancers increased by 75% between 2001 and 2021, and deaths increased by 55% with about 4,100 deaths in 2021 during the same period [28]. Between 2001 and 2021, approximately 72,000 people died in Germany due to skin cancer (melanoma and other malignant neoplasms of the skin) [29].

According to model calculations, a 1% reduction in stratospheric ozone could mean an increase in the incidence of malignant melanoma of 1 to 2% [30, 31], a 3 to 4.6% increase of squamous cell carcinoma, and a 2.7% increase for basal cell carcinoma [32-34]. Given the 3% ozone reduction that has occurred over Germany, this would theoretically mean an estimated increase of 3 to 6% for malignant melanoma, 9 to 15% for squamous cell carcinoma, and 9% for basal cell carcinoma. Assuming full compliance with the Montreal Protocol and taking skin type into account, modelling showed that three to four additional skin cancer cases per 100,000 inhabitants per year can be expected for Western Europe by the end of the 21^st^ century due to stratospheric ozone loss [35]. In Germany, with a population of about 83 million, this would result in approximately 2,500 to 3,300 additional skin cancer cases per year.

The LOEs observed in Germany lead to unexpectedly high UV irradiances, which are especially unexpected in spring, and sun protection measures are not considered accordingly. An associated higher sunburn risk and subsequently skin cancers can be assumed. However, quantification is currently not possible due to the lack of a description of a dose-effect relationship between individual UV dose and skin cancer incidence. The same applies to the quantification of the health impacts of an increased annual UV dose in years with plenty of sunshine.

The temperature increase associated with climate change is also discussed as a factor influencing skin cancer incidence. Studies show that heat stress can inhibit the programmed cell death of UV-damaged cells and that a temperature increase of 2°C could increase the incidence of non-melanoma skin cancer by about 11% [36–38]. The studies to date on the effect of higher temperatures on the development of UV-related skin cancer leave questions unanswered, so further research is needed to clarify and consolidate the findings [2].

The estimates of the increase in incidence found in the literature do not consider the behaviour of the population with regard to spending time outdoors – i.e. how long they expose themselves to which UV irradiances under which UV protection measures. This so-called UV exposure pattern is, however, a significant risk factor for skin cancer [2]. Sunburns as a consequence of intensive UV exposure, for example, double the risk of developing malignant melanoma – in children, the risk is reported to triple [2, 4].

Climate change-induced alterations in human sun exposure behaviour may have an important impact on future health risks from UV radiation. Previously published scientific studies support the theory that people spend more time outdoors in pleasant weather conditions and temperatures, wearing light clothing, and thus increase their individual UV exposure, especially under low cloud cover (radiant weather). A recent review summarises the scientific findings to date on climate-related changes in human sun exposure behaviour [39]. Accordingly, there are more opportunities for weather-dependent behaviour, especially in leisure activities and active leisure mobility, than in routine activities, even if the direction and extent of the effects vary. It also shows that it is not justified to transfer results and conclusions between different climatic zones and seasons and to generalise between different leisure activities and forms of active mobility. It should also be noted that behavioural changes may develop differently depending on individual human characteristics such as affinity for heat, leisure type, age, and gender. For a temperate climate, as prevails in Germany, the available studies provide indications that a possible increase in UV exposure would result primarily from a reduction in clothing and only secondarily from a change in the length of time spent outdoors. In the case of strong heat stress, there is a tendency to avoid spending time outdoors or in the sun. For Germany, the records of the Deutscher Wetterdienst (DWD; German Meteorological Service) show a clear increase in temperature, the number of so-called ‘summer days’ (temperature maximum at least 25°C) and ‘hot days’ (temperature maximum at least 30°C) compared to the reference period 1961 to 1990 [40]. Another article in this status report by Winklmayr et al. [41] provides detailed information on temperature development and heat in Germany. Ultimately, however, no quantitative statements about the individual UV exposure resulting from weather-dependent behaviour are possible at this time [2, 39].

## 4. Recommendations for reducing the risk of UV-related health damage

A nationwide, target group-oriented and sustainable establishment of suitable measures is urgently required due to the given situation with a high and currently increasing number of UV-related diseases in Germany and with the associated burden on the healthcare system and general welfare [2, 4, 42]. On top of that, there is the influence of climate change. Within the context of the ‘Climate Impact and Risk Assessment 2021 for Germany’, a medium to high climate risk with medium certainty for the period up to 2060 was determined for the climate impact of UV-related health damage and a very urgent need for action, in particular due to the long lead time [43]. Adaptation strategies to the health consequences of climate change should accordingly focus on preventive measures to prevent UV-related diseases [2]. Here it has to be emphasised that these measures do not mean avoiding UV radiation and thus the sun completely. Rather, it is a matter of establishing the conditions for a conscious approach to the sun and UV radiation, and of making liveable UV protection possible.

Suitable measures are primary prevention measures to prevent UV-related diseases [2]. Primary prevention comprises measures that, on the one hand, promote risk-conscious and health-oriented behaviour (behavioural prevention) and, on the other hand, shape people’s living, working, and environmental conditions in such a way that high UV exposures can be avoided to a large extent (structural prevention). In doing so, behavioural and structural prevention measures have to be meaningfully intertwined [44].

Effectively reducing the risk of UV-related diseases is a sociopolitical task and includes preventive measures from birth. In particular, an effective protection of children is required, as children have a significantly higher sensitivity of the eyes and the skin to UV radiation than adults [1].

Some measures, such as creating effective shaded areas, can simultaneously serve to prevent outdoor heat stress hazardous to health [45]. However, it must be considered that health-relevant UV irradiance levels can be present even when seeking shade is unnecessary, e.g. when it is cool or the sky is cloudy. Therefore, in places that are generally rather cool and where children as well as adults spend longer periods of time (outdoor areas in kindergartens and schools, public transport stops, etc.), it might be appropriate to install canopies from materials such as polycarbonate that block UV radiation but not heat. For shading measures, it should be noted that the sun-protective effect is influenced by the size, shape, and position of the shading structure, as well as by the surrounding environment and weather conditions [46]. Research suggests that shading of outdoor areas can increase the use of shade [2]. When using plants to generate shade, allergenic greenery should be avoided [43].

An important measure, in addition to creating shaded areas and ensuring UV protection for children, is to publicly display the prevailing UV irradiance, because UV radiation is not perceptible to humans and corresponding misconceptions of the UV-related health risk are ubiquitous. The sunburn-effective UV irradiance is measured by the Federal Office for Radiation Protection or modelled by the DWD and published as the UV Index, a globally uniform measure [47, 48]. The UVI values are divided into ranges for which different protection recommendations apply. The UVI is thus not only a guide for the expected sunburn-effective UV irradiance, but also provides recommendations as to the UVI values above which sun protection measures should be taken.

Recommendations with regard to surveillance and implications for a resilient public health system are addressed to science and politics (federal and state levels). With regard to their effective establishment and implementation, other actors are to be involved [42]. The recommendations listed in Table 1 are formulated in accordance with the recommendations of the oncological S3 guideline ‘Prevention of skin cancer’ [2] and the Climate Impact and Risk Assessment 2021 for Germany, sub-report 5 [43], and supplemented with further information and concrete measures for local authorities. Their order does not represent a weighting in terms of effectiveness or urgency. Unless otherwise stated, detailed information on the recommended measures can be found in the S3 guideline.

Effective implementation of the recommended measures also requires acceptance and motivation. The basis for this is, on the one hand, that medical, scientific, and official institutions provide comprehensive information regarding UV radiation, the effects of UV radiation, protection against UV radiation, preventive measures, and UV protection as a climate adaptation measure. On the other hand, funding is provided (see Info box 2).

## 5. Discussion and conclusion

The scientific measures considered necessary are very challenging – especially the description of a dose-response relationship for UV radiation/skin cancer and the projection of future ground-level UV exposure. Both the dose-response relationship and the UV projection are of great importance for a reliable assessment of the effects of future climate change-induced changes in UV exposure.

The measures recommended with regard to occupational and civil protection to reduce individual UV exposure are already partly being implemented. The driving force here is the fact that in 2015, due to the clear association with occupational UV exposure, the non-melanoma skin cancer entity, squamous cell carcinoma, and its precursors, actinic keratoses, were classified as an occupational disease [58]. Workers with outdoor jobs also have an increased risk of basal cell carcinoma [59]. Employers are accordingly obliged to carry out a risk assessment of outdoor activities, to take suitable protective measures [60] if necessary, and to document them. This applies to the approximately two to three million employees in Germany who work mainly or exclusively outdoors [61] as well as to children in day care centres and schools who are insured through accident insurances.

For an effective reduction of UV exposure for all, the measures recommended at the local level are crucial, which can also be seen in the context of recommendations in other articles of this status report, especially those on heat, as summarised by Winklmayr et al. [41]. At best, the recommended measures correspond or complement each other. However, with regard to the recommendations for adaptation to heat, one contradiction becomes apparent: for protection against UV radiation, surfaces of buildings should have low to no albedo (surface reflectivity) [2], but for heat reduction indoors, it is recommended to increase the albedo of building exteriors [43]. The recommendation for a low albedo is based on the fact that due to reflected radiation, UV exposure can be intensified in the vicinity of such buildings. A reduced surface albedo would also be positive in terms of heat protection, as the temperature near these buildings may increase due to a high reflectivity of building exteriors. One solution to this contradiction would be to achieve effective heat reduction indoors by means of appropriate building insulation and ventilation.

**Info box 2 Information, recommendations, and funding for effective UV protection in Germany**

► German Commission on Radiological Protection
► German Guideline Program in Oncology (German Cancer Society, German Cancer Aid, Association of the Scientific Medical Societies in Germany (AWMF)): S3 guideline ‘Prevention of skin cancer’
► German Federal Office for Radiation Protection: Background information and teaching materials on UV radiation, UV radiation and climate change, UV Index, effects of and protection against UV radiation, and on the UV campaign ‘UV safe’ with packages of measures for local authorities, kindergartens, schools, and sports clubs
► Federal Centre for Health Education (Bundeszentrale für gesundheitliche Aufklärung, BZgA): child health - sun protection for children and Klima-Mensch-Gesundheit (BZgA website, including the topics UV radiation and UV protection (2022))
► Climate Impact and Risk Assessment 2021 for Germany, sub-report 5, cluster ‘Health’, climate impact ‘UV-related health damage’
► Recommendations for the preparation of heat-health action plans including UV radiation
► German Cancer Aid (Stiftung Deutsche Krebshilfe) provides detailed information on its website about UV radiation and skin cancer
► Federal Institute for Occupational Safety and Health, German Social Accident Insurance and Federal State Accident Funds
► Funding by the Federal Ministry for the Environment, Nature Conservation, Nuclear Safety and Consumer Protection especially for municipalities and municipal institutions as well as social institutions

This compilation is a selection and makes no claim to completeness.

Behavioural and structural prevention measures to minimise UV-related disease risk are easy to implement, but can be cost-intensive. Internationally, such measures are evaluated positively from a health economic perspective, as corresponding analyses clearly showed a high economic and health-related benefit.

The investment can significantly reduce skin cancer cases and deaths, which are a particularly heavy burden on the health system, and save the health sector and the economy (due to prevented productivity losses) double to four times the costs invested in prevention measures [2].

The integration of preventive measures in municipalities including day care centres, schools, clubs, and societies requires a multidisciplinary approach. A shading policy established at the municipal level is recommended [46].

## Key statements

- UV radiation can cause serious skin and eye diseases, especially cancers.
- In Germany, UV irradiance and annual UV dose are changing and with them the individual UV radiation exposure.
- UV-related skin cancer incidences have been increasing for decades.
- The impact of climate change on the individual risk of skin cancer can currently only be predicted with considerable uncertainties.
- Learning and practising the right UV-protective behaviour starts at an early age and continues to be a necessity into old age.
- Measures to prevent UV-related diseases include synergistic effects with heat protection.
- The steady increase in incidence of UV-related skin cancer alone requires action be taken now, especially since it takes years to establish effective prevention measures.

## Figures and Tables

**Table 1:** Recommended actions for science and politics to reduce UV exposure and to prevent UV-related diseases Source: Own representation based on S3 guideline ‘Prevention of skin cancer’ [2] and Climate Impact and Risk Assessment [43]

Field of action	Recommendations
**Science**	► Quantification of the effects of climate change (greenhouse gases - ozone layer - low ozone events, cloud cover, and aerosols) on UV exposure ► Scientific evaluations and analyses of the current UV irradiance in Germany and the UV irradiance measured over the last decades. ► Development of suitable indicators for UV monitoring ► Development of projections of the UV radiation situation for Germany/Europe in relation to the scenarios modelled for possible future climate change developments, considering cloud cover, aerosol concentration, and reflectivity (albedo) ► Further research on the long-term effects of UV radiation on the eyes ► Establishment of registration procedures for UV-related diseases other than skin cancer and optimisation of existing registration procedures for all skin cancer entities ► Description of a dose-response relationship UV radiation/skin cancer ► Investigation of the influence of temperature on the effects of UV radiation ► Investigation of a possibly combined health effect of UV radiation, air pollutants, and meteorological factors ► Quantification of weather-dependent behavioural habits and clarification of the influence of climate change-induced weather and temperature changes on behavioural habits and individual UV exposure ► Evaluation of the implementation and effectiveness of recommended actions and preventive measures ► Development of geographic information system (GIS)-based modelling programmes to visualise UV exposure for urban and building planning as well as landscape architecture to create reasonably UV-reduced outdoor areas
**Occupational safety and health**	► Testing, evaluation, and optimisation of existing regulations and state rules on occupational health and safety (protection against work-related UV radiation hazards [49]) and on occupational medicine (precautionary examination for occupational disease BK 5103) in accordance with the Ordinance on Preventive Occupational Health Care (ArbMedVV) [50].
**Civil protection** Behavioural prevention measures	► Financial support for the nationwide establishment of behavioural prevention measures ► Pedagogically evaluated information and education about UV protection measures (e.g. [51]) and their necessity for children and adolescents in day care centres and schools, with the involvement of parents. Examples: ‘Clever in Sonne und Schatten’ (Clever in Sun and Shade) [52], SunPass program [53] ► Iterative, multi-media, interactive, multi-component, and multi-channel interventions for young people. ► Individualised interventions such as in the context of medical counselling sessions for adolescents and adults ► Integration of the topic ‘protection from UV radiation’ into teaching, educational, and training curricula for early childhood education and school teaching on the basis of a catalogue of basic principles, as well as into teaching, study, and training curricula and further and advanced training for the occupational profiles to be addressed ► Development, evaluation, and optimisation of information and training offers ► Creation and demand-driven dissemination of target group-oriented information materials for vulnerable population groups ► Support for the activities of education institutions regarding the conception of further education measures in the social, health, and care sector
**Civil protection** Structural prevention measures Federal / state levels	► Political and programmatic anchoring of behavioural and structural prevention measures for the prevention of UV-related diseases. The legal prerequisites for this are provided, in Germany, by the Prevention Act, the Early Cancer Detection and Registry Act, the ‘Patientenrechtegesetz’ (Act on Patients' Rights), supplemented by a legal obligation arising from the Building Code (§ 1, par. 6, nos. 1 and 7c; consideration of the general requirements for healthy living and working conditions as well as the environmental impact on people and their health) ► Inclusion of measures for the prevention of UV-related diseases in people’s living environments in funding programmes for adaptation measures to the impact of climate change and urban development funding ► Consideration of UV radiation as a health-relevant environmental factor in the establishment of an integrated environmental health monitoring system at federal level ► Establishment of preventive measures for the prevention of UV-related diseases within the framework of heat-health action plans [54] ► Integration of UV exposure into early warning systems (e.g. heat alert system) ► Information on characteristics of different tree species and on-site factors with regard to shade and transpiration processes for the effective reduction of heat and UV exposure ► Promoting the nationwide establishment of structural prevention measures at the municipal level
**Civil protection** Structural prevention measures Municipalities	► Identifying and establishing interfaces and docking points for structural prevention measures to reduce outdoor UV exposure in (planning) processes for urban development, town planning, and building planning. Embedding area-related activities to reduce health-relevant UV exposures in a cross-area development policy ► Inclusion of structural prevention measures, including the demand for their implementation in municipal tenders for urban development, urban and building planning as well as landscaping (new buildings, renovation measures of existing open spaces and buildings) ► Funding and municipal implementation of the following measures are recommended: ► Display of the UV Index in public spaces (outdoor swimming pools, bathing areas, city squares, etc.) ► Creation of shaded areas by means of structural-technical measures (roofing, sun sails, parasols, awnings, etc.) [55] as well as tree planting (see the road-side tree list of the German Conference of Garden Authorities [56]) in municipalities, in public transport waiting areas, and in day care centres and schools ► Use of non-reflective surfaces for building walls to reduce albedo (surface reflectivity) ► Construction of facade greening (see publication of the Federal Agency for Nature Conservation [57]) ► Desealing and greening of open spaces
